# Human hair follicles operate an internal Cori cycle and modulate their growth via glycogen phosphorylase

**DOI:** 10.1038/s41598-021-99652-8

**Published:** 2021-10-21

**Authors:** Katarzyna Figlak, Greg Williams, Marta Bertolini, Ralf Paus, Michael P. Philpott

**Affiliations:** 1 Centre for Cell Biology and Cutaneous Research, Blizard Institute, Barts & The London School of Medicine and Dentistry, Queen Mary University of London, 4 Newark Street, London, E1 2AT UK; 2 Farjo Hair Institute, London, UK; 3 Monasterium Laboratory, Münster, Germany; 4 Dr. Phillip Frost Department of Dermatology and Cutaneous Surgery, University of Miami Miller School of Medicine, Miami, FL USA; 5 Dermatology Research Centre, University of Manchester, and NIHR Biomedical Research Centre, Manchester, UK

**Keywords:** Cell biology, Insulin signalling, Nutrient signalling, Biochemistry, Polysaccharides

## Abstract

Hair follicles (HFs) are unique, multi-compartment, mini-organs that cycle through phases of active hair growth and pigmentation (anagen), apoptosis-driven regression (catagen) and relative quiescence (telogen). Anagen HFs have high demands for energy and biosynthesis precursors mainly fulfilled by aerobic glycolysis. Histochemistry reports the outer root sheath (ORS) contains high levels of glycogen. To investigate a functional role for glycogen in the HF we quantified glycogen by Periodic-Acid Schiff (PAS) histomorphometry and colorimetric quantitative assay showing ORS of anagen VI HFs contained high levels of glycogen that decreased in catagen. qPCR and immunofluorescence microscopy showed the ORS expressed all enzymes for glycogen synthesis and metabolism. Using human ORS keratinocytes (ORS-KC) and ex vivo human HF organ culture we showed active glycogen metabolism by nutrient starvation and use of a specific glycogen phosphorylase (PYGL) inhibitor. Glycogen in ORS-KC was significantly increased by incubation with lactate demonstrating a functional Cori cycle. Inhibition of PYGL significantly stimulated the ex vivo growth of HFs and delayed onset of catagen. This study defines translationally relevant and therapeutically targetable new features of HF metabolism showing that human scalp HFs operate an internal Cori cycle, synthesize glycogen in the presence of lactate and modulate their growth via PYGL activity.

## Introduction

Anagen hair follicles (HFs) have one of highest rates of cell division seen in any mammalian tissue with correlating high levels of protein synthesis due to ongoing formation of the keratinized hair fibre^1^. Although HF epithelial cells contain abundant functional mitochondria^2,3^ they preferentially engage in aerobic glycolysis metabolizing 90% of glucose to lactate despite the presence of oxygen^4,5^.

Although aerobic glycolysis is considered energetically inefficient compared to mitochondrial oxidative ATP synthesis (OXPHOS)^6,7^, cell metabolic needs extend beyond ATP production. Catabolism of glucose to lactate generate a high flux of metabolic precursors for biosynthesis, providing biomass accumulation^8,9^. HFs require energy and metabolite expenditure to maintain high proliferation rates, hair fibre production and hair cycle-associated HF remodelling. The importance of aerobic glycolysis to HF function has been demonstrated in mouse by the crucial role played by the enzyme lactate dehydrogenase and metabolism of glucose to lactate in the activation of epithelial HF stem cells (HFSC)^10^. Moreover, interplay between HF aerobic glycolysis OXPHOS and glutamine metabolism (glutaminolysis) functions as an important reversible switch between ORS lineage progression and their reversion back to HFSC^11^.

In a clinical context, excessive pathological production of lactate at the tissue level or impaired metabolism may cause lactic acidosis^12^. In the classical description of the Cori cycle, lactate from peripheral tissues such as the muscle is transported to the liver where it is converted via gluconeogenesis to glucose (Fig. 5b)^13–15^ which can be used to replenish glycogen (a branched glucose storage polymer) and protect tissues from hyperlactatemia^16,17^.

Glycogen permits osmotically neutral storage of energy and carbon^18,19^ (Fig. 5a). Glucose-6-phosphate (G6P) units released from glycogen degradation^20^ are metabolized through glycolysis to generate ATP and lactate/pyruvate or through the pentose phosphate pathway (PPP), producing NADPH and biosynthesis precursors^21^. The ORS (outer root sheath) of HFs is reportedly rich in glycogen^22–24^. A role for glycogen in HF physiology has long been proposed both with regards keratinization^23^ and hair cycle regulation in mice^25^ and humans^24^.

We hypothesize that as HFs engage in aerobic glycolysis^10,11,26^, generated lactate may be utilized by the ORS and metabolized to glycogen via an internal HF/ORS Cori cycle, explaining the high levels of glycogen reported in the ORS^22^. We also asked whether ORS glycogen was available for HF metabolism and whether it played a role in HF function.

To investigate this we have made use of isolated human HFs, a unique mini-organ model that can be cultured ex vivo where it continues to engage in aerobic glycolysis and glutaminolysis^5^ maintaining in vivo rates of keratinocyte proliferation, differentiation and hair fibre synthesis^5,27^ responding physiologically to a wide range of growth regulatory factors and hormones^28,29^. We now demonstrate functional glycogen synthesis and catabolism as well as the presence of an operative human HF Cori cycle. Further we show that inhibition of glycogen phosphorylase (PYGL) prolongs anagen maintenance and hair growth. Together these data show that HFs can utilize lactate from aerobic glycolysis to generate glycogen and that metabolism of glycogen plays an important role in human hair cycle regulation.

## Results

### Glycogen content changes during the human hair cycle

**Figure 1:**
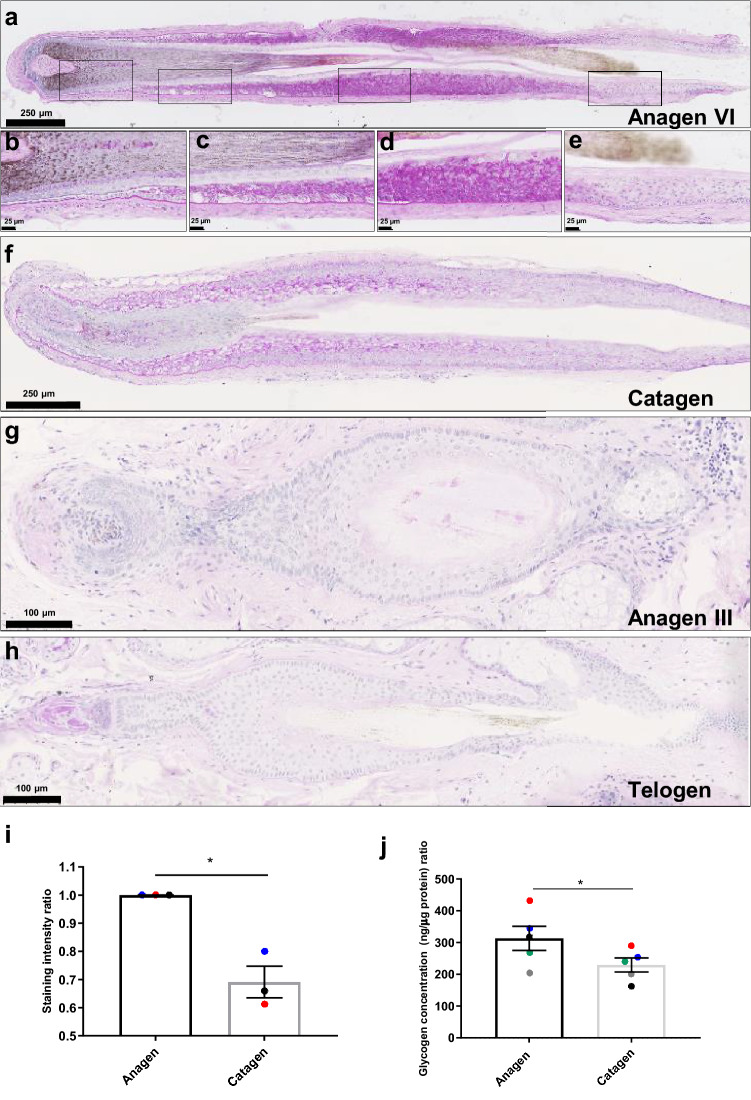
Glycogen content changes within the hair cycle. PAS staining of human HFs in anagen VI (**a**–**e**), showing various compartments of anagen follicles (**d**–**e**); catagen (**f**) early anagen (**g**) and telogen (**h** Data represents staining on hair follicles isolated from 3 separate individuals (N = 3) using 3–4 HFs from each individual. Only two telogen and one early anagen HF were detected through serial sections of 3 skin biopsies. (**i**) Intensity quantification of the ORS of PAS-stained ORS in anagen and catagen HFs, measurement was made on hair follicles from 3 separate individuals (N = 3) using 3–4 anagen and catagen HFs per sample in every individual * p < 0.05, paired t-test,  ±SEM. (**j**) Glycogen content quantified by the enzymatic colorimetric glycogen assay in whole anagen and catagen HF’s. Measurements were made on HF lysates from 5 separate individuals (N = 5) using the same number of HF’s (3–4 HFs per individual) for each anagen and catagen lysate. * p < 0.05, paired t-test,  ±SEM. Positive controls were carried out using PAS stained liver and negative controls were carried out using 0.5% porcine α-amylase treatment of liver and human skin to confirm specificity of PAS staining, see Materials & Methods and Supplementary Fig. 1.

In human anagen VI HFs strong intraepithelial Periodic-Acid Schiff (PAS) staining for glycogen^30,31^ was detected in the suprabasal cells of the bulbar ORS (Fig. 1a,b) and the basal and suprabasal cells in the suprabulbar ORS, adjacent to keratinized inner root sheath (IRS (Fig. 1a,c-d). Glycogen diminished towards the isthmus, and was not detected in the HFSC niche/bulge^32^ (Fig. 1e). Glycogen was detected in presumptive hair shaft cuticle cells from Auber’s line to the zone of cuticle keratinization (Fig. 1b-d) with weak presence in the HF medulla (Fig. 1a,b). Glycogen was not detected in the hair matrix (HM), cortex or the IRS (Fig. 1a–c). 0.5% porcine α-amylase treatment of skin and liver was used as negative control to confirm specificity of PAS staining for glycogen (Supplementary Fig. 1).

HF glycogen fluctuates during the murine hair cycle^25^. Glycogen also markedly decreased in ex vivo human anagen VI HFs undergoing spontaneous catagen^33^ (Fig. 1f) with especially pronounced loss of glycogen in basal cells of the suprabulbar ORS. Glycogen quantification of PAS histomorphometry in the ORS (Fig. 1i) and colorimetric quantitative glycogen assay^34^ on whole HF lysates (Fig. 1j) confirmed a significant catagen-associated decrease in glycogen compared to anagen HFs (*P* < 0.05). Telogen (Fig. 1g) and anagen III (Fig. 1h)^35^ HFs, with the exception of occasional weakly positive cells in the secondary hair germ, contained no glycogen. This is intriguing, since it further consolidates suggestions that telogen HFs, especially the secondary hair germ, are by no means “quiescent”, not only at the level of gene and protein expression^36,37^ but also metabolically.

### Key enzymes of glycogen synthesis and catabolism are expressed in the HF outer root sheath

**Figure 2:**
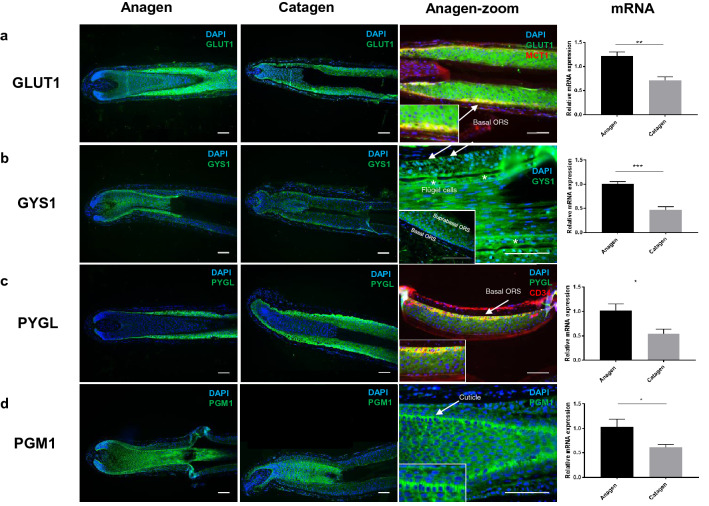
HFs express crucial glycogen metabolism proteins and actively uptake and accumulate glucose. GLUT1 (**a**), GYS1 (**b**), PYGL (**c**) and PGM1 (**d**) immunofluorescence in cultured anagen and catagen human HFs. Details of suprabulbar anagen HFs (**a**) GLUT1 (green) and MCT1 (red), arrow-basal ORS cells; (**b**) GYS1 (green) showing basal and suprabasal ORS and IRS expression, arrow-basal ORS-KC, Flügel cells (asterisk). (**c**) Suprabasal ORS stained with PYGL (green) and CD34 (red), arrow-basal ORS. (**d**) PGM1 (green), arrow-hair cuticle cells. Representative images of at least 12 images from 5 individuals. Scale bars 100 μm. mRNA expression of GLUT1 (**a**) GYS1 (**b**) PYGL (**c**) and PGM1 (**d**) in whole HFs cultured in vitro for 1 (anagen) and 6–7 days (catagen). 5 HFs per sample, N = 4, normalized to B2M. Paired two-tailed t-test (*p < 0.05, **p < 0.005),  ±SEM.

Using IF microscopy and qPCR we investigated whether human HFs express the key transporters and enzymes essential for glucose uptake, glycogen synthesis and catabolism^38^. As shown in Fig. 2 and summarized schematically in Fig. 5c, GLUT1, GYS1, PYGL, and PGM1 protein and mRNA were all expressed in the ORS of human scalp HFs. Quantification on mRNA and protein (western blot densitometry) were carried out but while mRNA (Fig. 2) showed similar levels between patient’s protein levels showed marked interpatient variability (Supplementary Fig. 2) and did not reflect the decrease in mRNA expression; probably reflecting the longer half-life and slower turnover of metabolic enzymes.

*Glucose transporter 1* (GLUT1) the major glucose transporter in human cells^39^ was strongly expressed in the ORS of anagen HF (Fig. 2a) co-aligned with MCT1 (monocarboxylate transporter 1) (Fig. 2a, arrow). In catagen HFs, GLUT1 mRNA but not protein was significantly (*P* < 0.01) downregulated (Fig. 2a and Supplementary Fig. 2).

*Glycogen synthase 1* (GYS1) crucial in the conversion of glucose into glycogen (Fig. 5a)^40^. In anagen HFs GYS1 was strongly expressed in the basal cells of the ORS (Fig. 2b) as well as in the suprabasal ORS and non-keratinized IRS. GYS1 expression was also clearly visible in the highly specialized IRS Flügel or winged cell (Fig. 2b-asterisks)^41^. Low levels of GYS1 were seen in the hair matrix, pre-cortex, suprabasal ORS-KC as well as the connective tissue sheath and DP. In catagen HFs GYS1 remained highly expressed in basal keratinocytes of the ORS with weak expression in the hair bulb and developing club hair. The fully keratinized IRS was negative for GYS1 (Fig. 2b). GYS1 mRNA but not protein was significantly downregulated in catagen HFs (Fig. 2b and Supplementary Fig. 2).

*Glycogen phosphorylase* (PYGL) co-localizes within the same region of the ORS in anagen HFs as glycogen (Fig. 2c, compare with Fig. 1b) with the strongest expression in suprabulbar region disappearing gradually towards bulge (Fig. 2c). Glycogen phosphorylase was strongly expressed and co-localized to CD34 positive basal cells of the ORS (Fig. 2c, arrow), similarly to GYS1. In catagen HFs PYGL mRNA but not protein was significantly downregulated in the ORS (Fig. 2c and Supplementary Fig. 2).

*Phosphoglucomutase 1* (PGM1) is involved both in glycogen synthesis and degradation^42^. In anagen HF strongest PGM1 IF signal was detected in the cuticle (Fig. 2d, arrow), with weaker expression in the bulbar and suprabulbar ORS, IRS, HM and pre-cortex. Weak staining for PGM1 was also observed in the upper part of the dermal papilla (Fig. 2d). In catagen follicles PGM1 was detected in the DP, with stronger staining in the HM, precortex and cuticle up to the point of full keratinization, after which expression abruptly disappeared (Fig. 2d). PGM1 mRNA but not protein was significantly (*P* < 0.05) downregulated in catagen HFs (Fig. 2d and Supplementary Fig. 2).

### Human ORS-KC engage in a functional Cori cycle and glycogen shunt

HFs metabolize the majority of their glucose to lactate^5,43^. Lactate generated by peripheral tissues enters the bloodstream and is transported to the liver where it is converted to glucose and glycogen via the gluconeogenesis completing the Cori cycle. Having shown that the ORS contains high levels of glycogen and key enzymes of its metabolism, we asked whether HFs operate their own internal Cori cycle and can reuse lactate to generate glycogen via gluconeogenesis and glycogen synthesis (Fig. 5b).

**Figure 3:**
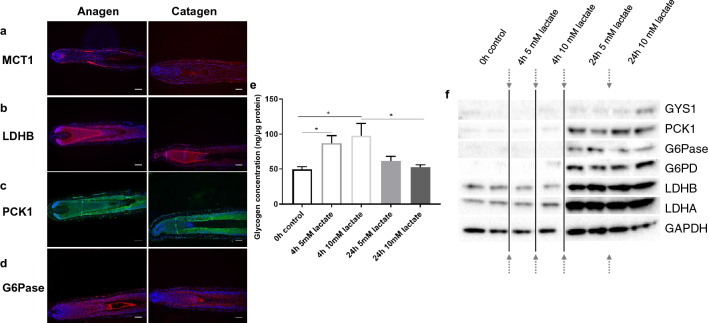
HFs express key enzymes of gluconeogenesis. ORS-KC and HFs engage in gluconeogenesis and glycogen shunt. Immunofluorescence staining of anagen and catagen HFs for MCT1 (**a**), LDHB (**b**), PCK1 (**c**) and G6Pase (**d**). Scale bar 100 μm, representative images of 9 images from 3 individuals. (**e**) Glycogen in ORS-KC with 5 and 10 mM lactate Mean ±SEM. Paired two-tailed t-test, * p < 0.05. (**f**) Western Blot of GYS1, PCK1, G6Pase, G6PD, LDHB and LDHA in ORS-KC; after 4 and 24 h in 5- and 10-mM lactate. control GAPDH, arrows representing cropped images, representative N = 3 experiments.

Immunofluorescence showed that the key gluconeogenesis enzymes and transporters; lactate transporter MCT1 (Fig. 3a), lactate dehydrogenase B (Fig. 3b), phosphoenolpyruvate carboxykinase 1 (Fig. 3c), and glucose-6-phosphatase (Fig. 3d)^15^ were expressed in the ORS and hair shaft cuticle of anagen HFs, similarly to glycogen and the enzymes of glycogen metabolism (Fig. 5c), suggesting that human scalp HFs are equipped with the complete enzymatic machinery for gluconeogenesis. To investigate this functionally we used cultured primary human ORS keratinocytes (ORS-KC)^44,45^ as an accessible model to investigate whether lactate promoted gluconeogenesis. Glycogen concentration in ORS-KC significantly increased after 4 h of incubation with lactate and returned to control values after 24 h (Fig. 3e) indicative of active glycogen synthesis and a glycogen shunt^46^.

In support of our data showing increased ORS glycogen synthesis in response to lactate Western Blot analysis of ORS-KCs incubated for 24 h with lactate (Fig. 3f) showed marked up-regulation of gluconeogenic enzymes including rate limiting enzymes PCK1, G6Pase as well asG6PD, LDHA, LDHB and GYS1 in response to lactate, further suggesting active pathways of gluconeogenesis and glycogen synthesis.

### PYGL regulates glycogen breakdown in Human ORS-KC

**Figure 4:**
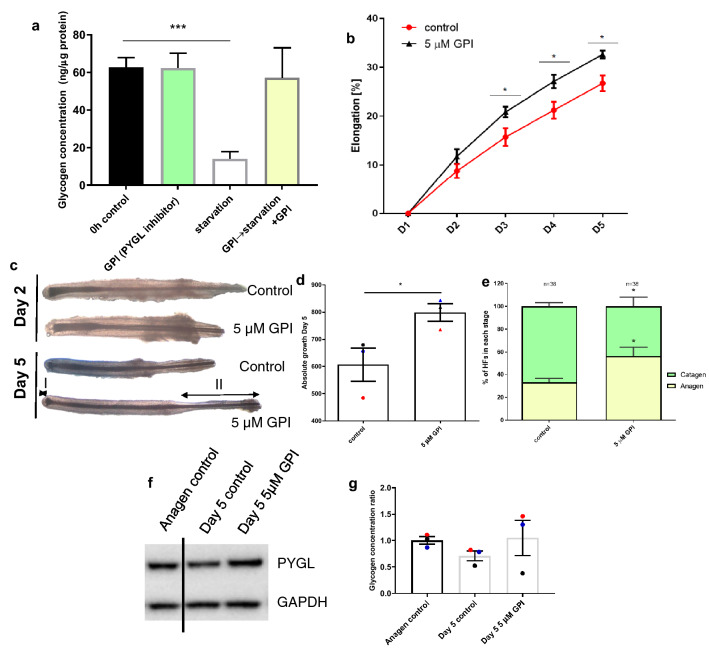
PYGL inhibition stimulates HF growth. (**a**) Glycogen assay in primary ORS-KC lysates showing that ORS-KC can utilise glycogen under starvation conditions and that utilisation is blocked by the specific PYGL inhibitor GPI. N = 3. (**b**) Effects of 5 µM GPI on ex vivo HF elongation after 5 days, N = 5. (**c**) HF morphology in control and GPI samples after 1 and 5 days, N = 5 individuals. (**d**) Absolute HF elongation after 5 days ex vivo, N = 5. (**e**) Hair cycle staging assessed in control and GPI treated HFs after 5 days of culture, staging carried out on N = 38 HFs from 3 donors. (**f**) Western Blot analysis of PYGL expression. GAPDH used as a loading control. Representative image of N = 3 experiments (Supplementary Fig. 3). (**g**) Glycogen assay performed in HF lysates. 3–4 HFs per sample, N = 3. Mean ±SEM. One-way ANOVA (**a**,**g**); two-way ANOVA (**c**) paired two-tailed t-test (**d**,**f**). * p < 0.05, ** p < 0.01, *** p < 0.001.

Since glucose and glutamine are crucial metabolic fuels for human HF growth ex vivo and key precursors of glycogen synthesis^26^, we next asked if glycogen stores are being actively depleted upon glucose and glutamine starvation, and if glycogen breakdown in ORS-KC is regulated by PYGL. Quantitative glycogen assay showed that human primary ORS-KC cultured in serum-free medium lacking both glucose and glutamine had significantly depleted glycogen stores (Fig. 4a) Using a PYGL specific glycogen phosphorylase inhibitor (GPI)^47^ we showed that in glucose and glutamine starved ORS-KC 5 µM GPI significantly inhibited glycogen breakdown, indicating that PYGL is the crucial glycogen metabolism enzyme in human ORS-KC.

### PYGL inhibition stimulates human hair growth ex vivo

To investigate the impact of PYGL on HF growth, organ-cultured HFs^29^ were incubated in serum-free supplemented medium containing 5 µM GPI. The ex vivo culture of isolated human HF is a well-established method for investigating factors that regulate the growth of human hair follicles (see^28,29^ for extensive reviews of the model). HFs maintained with GPI showed a statistically significant increase in overall hair follicle elongation over controls (Fig. 4b,c), specific measurement of the hair fibre during this time confirmed the increase in hair follicle elongation was due to increased hair shaft formation over controls (Fig. 4d). Most importantly, treatment with GPI significantly prolonged anagen while having no significant effect on PYGL expression (Fig. 4e,f and Supplementary Fig. 3). In contrast, control HFs maintained in culture for 5 days demonstrated morphological signs of early catagen (see review for key morphological changes^28^) and showed trends towards decreased glycogen levels compared to anagen control HFs although the effects of GPI on glycogen were mixed with 2 patients follicles showing strong response to GPI inhibition of glycogen metabolism but follicles from the third patient appearing to be unaffected (Fig. 4g).

This data constitutes the first evidence in the literature that inhibiting glycogen catabolism profoundly impacts on human hair shaft production and HF cycling. This is in line with the observation that HF glycogen content declines during catagen development (Fig. 1g,j) and suggests that catagen development is a highly energy-consumptive process that requires substantial glycogenolysis to proceed; vice versa, maintaining high glycogen levels in the ORS counteracts this and prolongs active hair growth (anagen).

## Discussion

Glycogen has previously been reported in the ORS of both human and murine HFs^22,48^. We confirmed and extended these studies to include glycogen quantification, localization of the key enzymes of glycogen metabolism and glucose synthesis within HF compartments and the human hair cycle. Importantly we demonstrated a functional Cori cycle and showed that glycogen metabolism was important for human HF growth and cycling.

Human HFs expressed the crucial enzymes of glycogen metabolism and these correlated with highest concentrations of glycogen-mainly in the ORS, but also the cuticle and pre-cortex. Adachi and Uno^49^ described activity of the enzymes of glycogen metabolism in the suprabulbar HF although these studies were incomplete as the authors used plucked HFs and plucking damages the HF leaving the hair matrix and DP in the skin.

The highest concentration of glycogen observed in the suprabasal ORS correlated with high expression of rate-limiting glycogen metabolism enzymes, especially GYS1 and PYGL responsible for glycogen synthesis and breakdown. PYGL and GYS1 also co-localized with a population of CD34 positive cells. CD34 has been identified as a putative marker of epithelial HF stem cells (HFSCs) and distinct from the established bulge or stem cells region of human HFs^32,50^. Glycogen was not detected in the bulge, suggesting glycogen presence is not linked with all HFSC niches and CD34+ cells containing glycogen may present a specific stem cell population with different metabolic regulation to other HFSCs. CD34+ cells were reported to markedly diminish in scalps of patients with androgenetic alopecia, whereas the KRT15+ stem cells were maintained^51^; it would therefore be of interest to know the glycogen status of balding HFs and whether loss of CD34+ cells correlated with changes in ORS glycogen.

The human HF is known to engage in aerobic glycolysis and to metabolize most of its glucose to lactate^5^. The activity of lactate dehydrogenase and lactate production has been shown to drive the activation and maintenance of bulge HFSCs in mice, suggesting that metabolism acts as a stimulus in the switch between dormancy and proliferation^10^. Lactate generated by glycolysis in muscles is transported to the liver and where through gluconeogenesis lactate can be converted to glucose and replenish glycogen^13–15^. We showed that under conditions of nutrient starvation ORS-KC rapidly metabolized glycogen catalyzed by glycogen phosphorylase (PYGL). This demonstrated that glycogen can be utilized by the ORS and may be used as an energy store by the HF.

Since HF metabolize the majority of their glucose to lactate^5,43^ and the ORS contains all of the enzymes for glucose and glycogen synthesis, we asked whether HFs had an internal Cori cycle and could use lactate to generate glycogen. ORS-KC incubated with lactate significantly up-regulated glycogen levels and gluconeogenic enzymes suggesting that ORS-KC engage in gluconeogenesis and can synthesize glucose and glycogen from lactate and therefore operate an internal Cori cycle. This would allow HF to take up locally produced lactate for the synthesis of glycogen. We observed that the increase in glycogen in response to lactate was transient indicative of the glycogen shunt in which glucose is incorporated into glycogen and upon metabolic demand, enters glycolysis via phosphorylase rather than directly entering glycolysis, coupling glycogen synthesis and breakdown to glycolysis^46^.

The glycogen shunt is frequently associated with tissues and cells that engage in aerobic glycolysis and allows cells to store glucose as glycogen for future use while maintaining homeostasis of glycolytic intermediates and ATP^52^. In memory CD8+ T cells, G6P is channelled to glycolysis and to PPP to generate NADPH^53^. We also observed upregulation of G6PD, a key enzyme of PPP upon the treatment with lactate, suggesting similar mechanism occurring in the ORS.

Although the ORS contains high levels of glycogen it is not known whether this serves any function role-possibly as a HF energy store. To investigate this with regards human HF growth we cultured isolated human HF ex vivo^27,28^ with a glycogen phosphorylase inhibitor (GPI). We used GPI to inhibit PYGL activity rather than siRNA knockdown on mRNA as we found in catagen HF that despite significant decrease in mRNA enzyme (protein) expression remained stable; possibly reflecting the long half-life of metabolic enzymes; moreover, the concentration of siRNA required to knockdown PYGL was toxic to the HF (not shown). GPI significantly stimulated HF growth which correlated with hair fibre production and significantly reduced the number of HFs entering catagen.

**Figure 5:**
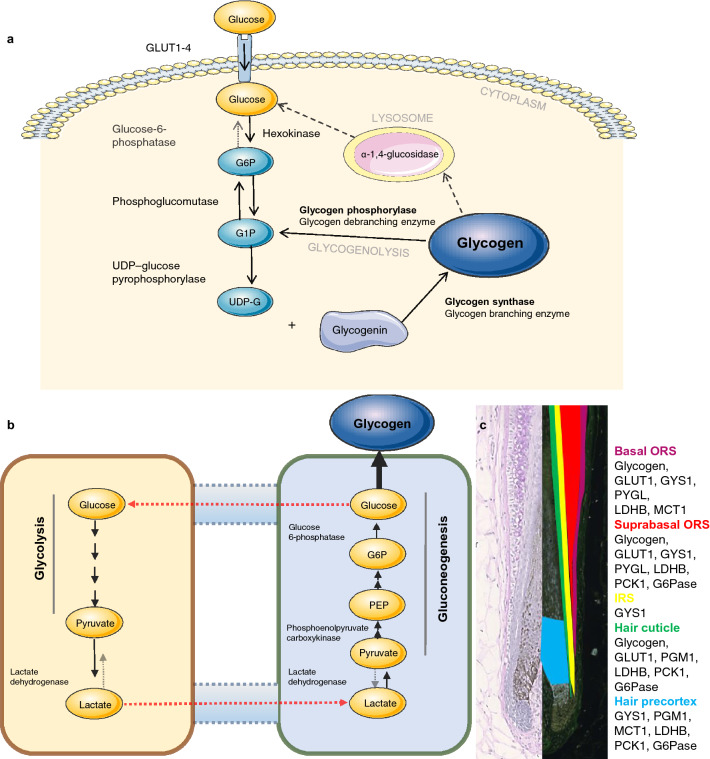
Schematic overview of glycogen metabolism and Cori cycle. (**a**) Intracellular glycogen synthesis and breakdown including rate-limiting enzymes glycogen synthase (GYS) and glycogen phosphorylase (PYGL). (**b**) Cori cycle-gluconeogenesis is reverse process of glycolysis with exception for reactions catalyzed by rate-limiting enzymes of gluconeogenesis: PCK, FBPase and G6Pase. G6P-glucose-6-phosphate, G1P-glucose-1-phosphate, UDP-G-UDP-glucose. (**c**) Cartoon summary of bulbar and suprabulbar anagen HF with localization of glycogen and key proteins of glycogen metabolism and gluconeogenesis in the HF epithelium. Figures (**a**) and (**b**) were created using Servier Medical Art templates, which are licensed under a Creative Commons Attribution 3.0 Unported License; https://smart.servier.com.

The mechanism by which inhibition of PYGL stimulated HF growth ex vivo and reduces catagen is probably complex. Catagen is driven by apoptosis and apoptosis requires energy^54^. our data showing that inhibition of by GPI significantly reduced the number of HF entering catagen suggest that PYGL activity is important for catagen. As the only known action of PYGL is glycogen breakdown this data suggest that glycogen metabolism is essential for catagen possible supplying the energy required for apoptosis. However, while measurement of HF glycogen showed an overall trend towards increased glycogen in GPI treated follicles (Fig. 4g) one patient showed decreased glycogen. This patients HF may have been non-responsive to GPI however, further work is required to establish a direct relationship between glycogen and catagen (Fig. 5). We have not directly measured apoptosis in our follicles but others have shown increased apoptosis in spontaneous catagen HF ex vivo^28^. More speculative, glycogen is known to inhibit AMPK^40^. AMPK is an important metabolic sensor and under conditions of metabolic stress AMPK inhibits aerobic glycolysis allocating nutrients toward catabolic/energy-producing and away from anabolic/growth-promoting metabolic pathways^55^. Whether glycogen accumulation in the HF inhibits AMPK allowing the HF to engage in aerobic glycolysis and towards anabolic growth promoting pathways remains to be determined. However, our data suggest that glycogen is a marker of active hair growth.

Finally; glycogen has been proposed to play a role in keratinization^23^. The main sites of expression of glycogen metabolism proteins are suprabulbar ORS, hair cuticle and precortex, and the accumulation of glucose in these regions may reflect HF compartments with the highest metabolic rates^3^. Co-aligned expression of the enzymes involved in glycolysis (PGM1, LDHA, LDHB) in the cuticle and the pre-cortex, together with the transient glucose accumulation suggest that they be may be regions of enzyme activity directed towards rapid keratinization and adapted to quick metabolic changes. Finally, the ORS could probably act as a stable glycogen storage site used both for anagen maintenance and remodelling and regression in catagen. This later role is suggested by our data showing that inhibition of PYGL by GPI significantly reduced the number of HF entering catagen suggesting that glycogen breakdown is essential for catagen but also despite significant decrease in mRNA expression in catagen, protein levels for the enzymes did not change significantly.

In conclusion, we have shown that the HF ORS is a major site of glycogen synthesis and functional glycogen storage and is capable of gluconeogenesis. That inhibiting glycogen catabolism ex vivo promotes hair growth and delays catagen, prolonging anagen. This constitutes a promising target for future research with regards HF nutrition and nutrient sensing.

## Materials and methods

### Human HF collection and organ culture

HF units from occipital region were obtained from hair transplant patients and whole scalp skin samples from face lift patients following ethical approval granted by East London and The City Research Ethics Committee 1 (REC Alpha 09/H0704/40) and written informed patient consent, adhering to the Declaration of Helsinki Principles. All samples, slides and biological material were tracked and stored according to the ‘Human Tissue Act’ guidelines. Anagen VI HFs were microdissected and cultured as previously described^27^. ORS-KC were isolated as previously described^56^ with a modification-freshly isolated ORS-KC were seeded in collagen I (Thermo Fisher, UK) coated flasks and cultured in Keratinocyte Serum Free Medium (Thermo Fisher, UK). Starvation was performed by incubation in SILAC RPMI 1640 Flex Medium (Thermo Fisher, UK). PYGL inhibitor (Cayman Chemicals, UK) and 2-NBDG (Thermo Fisher, UK) were diluted from stock DMSO solution in culture medium. Lactate (Sigma-Aldrich, UK) was diluted in culture media.

### RNA extraction, qPCR analysis

Total RNA was extracted from ORS-KC and HFs using the Qiagen RNeasy Microkit (Qiagen, UK) following the manufacturer’s instructions. cDNA was synthesized using SuperScript VILO cDNA Synthesis Kit (Invitrogen, UK). qPCR was performed using Rotor-Gene SYBR Green PCR Kit (Qiagen, UK). Relative expression was determined against B2M-Beta-2-microglobulin.

### Periodic Acid Schiff staining

Formalin-fixed paraffin-embedded (FFPE) sections were stained using Periodic Acid Schiff (PAS) Kit (Sigma-Aldrich, UK), according to manufacturer’s protocol. Negative control sections were digested using porcine α-amylase (Sigma-Aldrich, UK). Sections were imaged using Nanozoomer slide scanner (Hamamatsu, Japan). PAS quantification was performed using ImageJ software, intensity of staining was calculated in the ORS of HFs and normalized to cell number.

### Immunofluorescence

Antigen retrieval was performed on 5 µm FFPE tissue sections using Citrate buffer, primary antibodies (GYS1, PGM1, LDHB-Abcam, UK; PYGL, Glut1, PCK1-Proteintech, UK; MCT1-Santa Cruz, UK; CD34-BD Biosciences) were incubated overnight at 4 °C followed by appropriate Alexa Fluor secondary antibodies (Thermo Fisher, UK) and DAPI for nuclear staining. Analysis of immunostaining were performed using DM5000B (Leica) and LSM880 (Zeiss) microscopes.

### Glycogen concentration assay

Glycogen was quantified using a coupled enzyme colorimetric Glycogen Assay Kit (Sigma-Aldrich, UK) according to manufacturer’s protocol, normalized to protein concentration [Pierce BCA Protein Assay Kit (Thermo Fisher, UK)].

### Western blotting

ORS-KCs and HFs homogenized in RIPA buffer containing protease and phosphorylase inhibitors (Sigma-Aldrich, UK) were separated using NuPAGE minigels (Invitrogen, UK) according to the manufacturer’s instructions then transferred to a Trans-Blot Turbo PVDF membrane (Bio-Rad, UK) and incubated overnight at 4 °C with primary antibodies (β-Actin, G6Pase, G6PD, LDHA-Santa Cruz, UK; GYS1, Glut1, PCK1, PYGL-Proteintech, UK; GAPDH, LDHB, PGM1-Abcam) and thereafter with appropriate secondary horseradish peroxidase-conjugated antibodies (Dako, Denmark). Membranes were imaged using Clarity ECL chemiluminescence solution (Bio-Rad, UK) and visualized using ChemiDoc XRS (Bio-Rad, UK).

### Statistical analyses

Biological replicates and number of HFs used are indicated in the respective figure legend. Statistical was carried out using either two-tailed paired t-test, one-way ANOVA or two-way ANOVA with multiple comparisons. Data was expressed as mean ± SEM and considered statistically significant at a *P* value < 0.05.

## Supplementary Information

Supplementary Information 1.

Supplementary Information 2.

## Data Availability

All relevant data are included in the manuscript. Used datasets are available from the corresponding author on reasonable request.

## Abbreviations

CTS: Connective tissue sheath
G6Pase: Glucose-6-phosphatase
GLUT1: Glucose transporter 1
GPI: Glycogen phosphorylase inhibitor
GYS1: Glycogen synthase 1
HF: Hair follicle
HM: Hair matrix
IRS: Inner root sheath
LDHB: Lactate dehydrogenase B
ORS: Outer root sheath
ORS-KCs: Outer root sheath keratinocytes
PAS: Periodic Acid Schiff’s
PCK1: Phosphoenolpyruvate carboxykinase 1
PGM1: Phosphoglucomutase 1
PPP: Pentose phosphate pathway
PYGL: Glycogen phosphorylase
