# Supplementary material for: Human hair follicles operate an internal Cori cycle and modulate their growth via glycogen phosphorylase
Source: Sci Rep. 2021 Oct 21;11:20761. doi: 10.1038/s41598-021-99652-8 (PMC8531296; doi:10.1038/s41598-021-99652-8)
Supplement: Supplementary file 1 — Supplementary Information 1. [file 41598_2021_99652_MOESM1_ESM.pdf]

Original Western Blot membrane images

Human hair follicles operate an internal Cori cycle and modulate their growth via glycogen phosphorylase

Katarzyna Figlak[1], Greg Williams[2], Marta Bertolini[3], Ralf Paus[3,4,5] and Michael P Philpott[1]*

[1]Centre for Cell Biology and Cutaneous Research, Blizard Institute, Barts & The London School of Medicine and Dentistry, Queen Mary College University of London, UK; [2]Farjo Hair Institute, London, UK; [3]Monasterium Laboratory, Münster, Germany; [4]Dr. Phillip Frost Department of Dermatology and Cutaneous Surgery, University of Miami Miller School of Medicine, Miami, FL, USA; [5]Dermatology Research Centre, University of Manchester, and NIHR Biomedical Research Centre, Manchester, UK

Raw images of western blot membrane from Figure 3f Part 1

The membrane was cut at 50kD to include more analysed proteins-the antibodies were optimised before the experiments and did not demonstrate unspecific binding

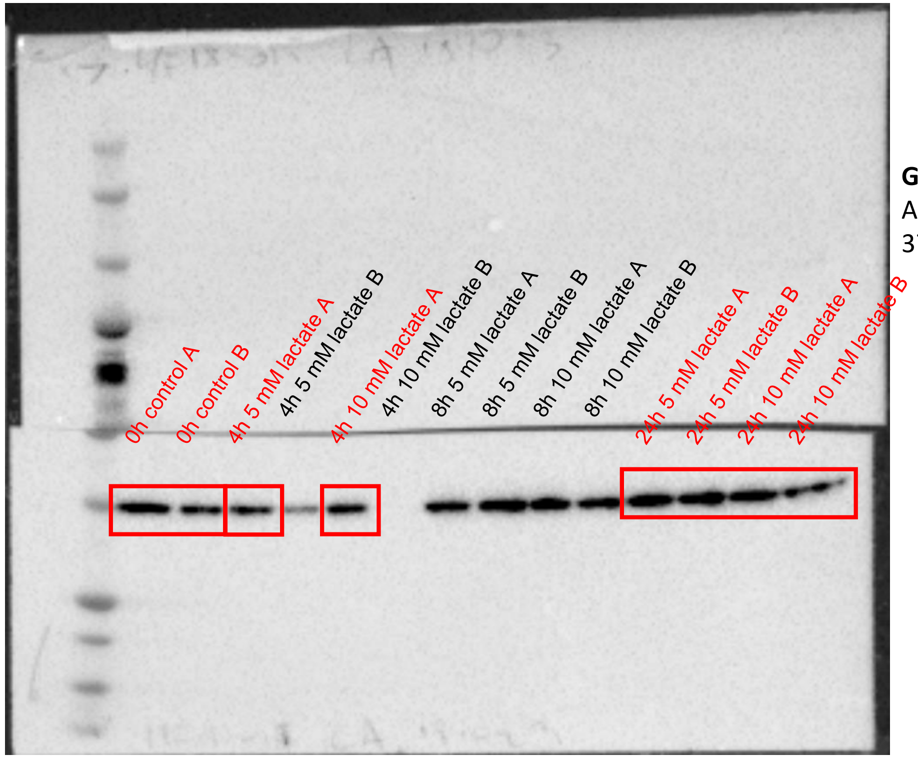

GAPDH, ab9485
Abcam
37 kD

Precision Plus Protein
All Blue Protein
Standard
Bio-Rad 1610373

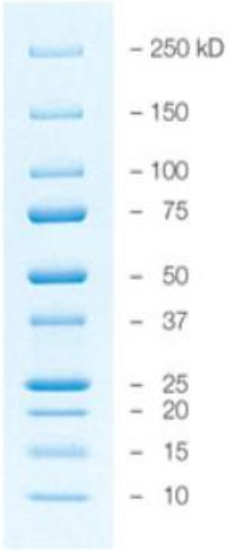

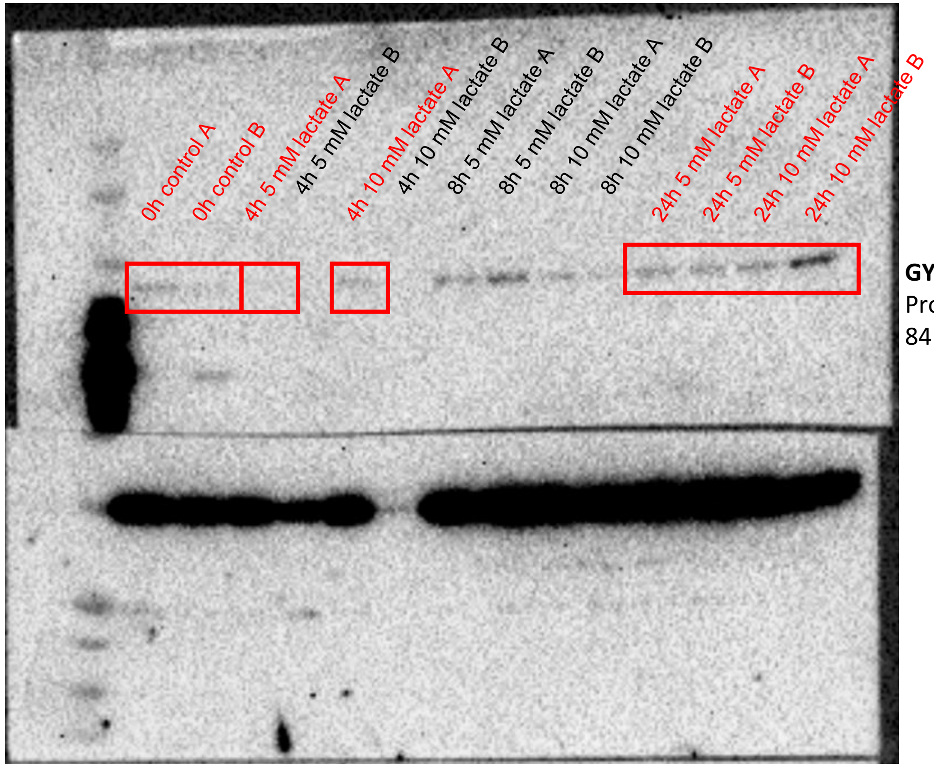

GYS1, 10566-1-AP
Proteintech
84 kD

Raw images of western blot membrane from Figure 3f Part 2

The membrane was cut at 50kD to include more analysed proteins-the antibodies were optimised before the experiments and did not demonstrate unspecific binding

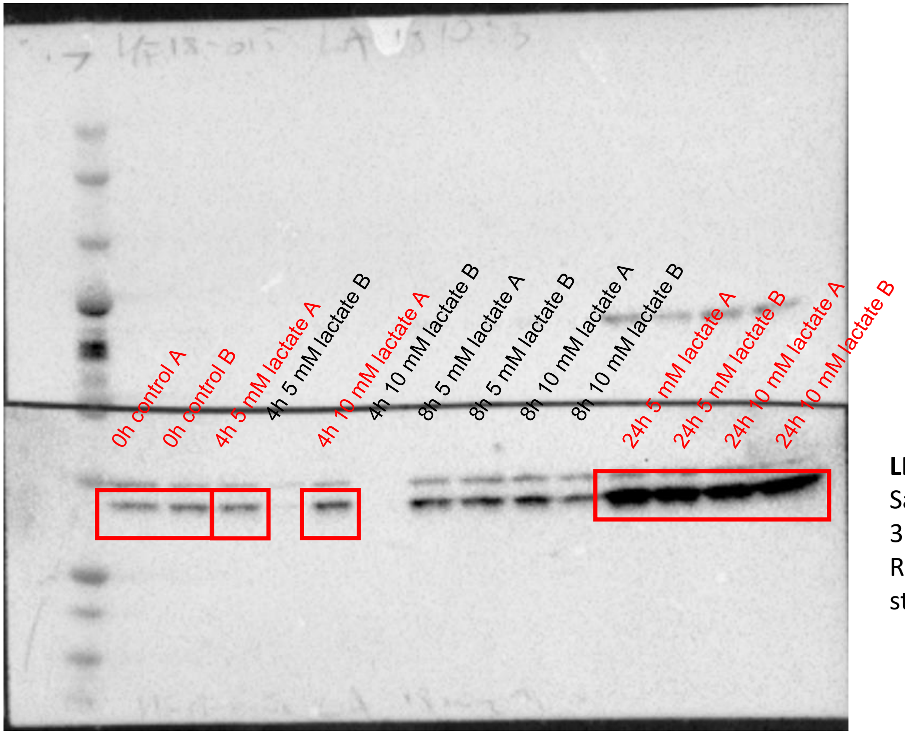

LDHA, sc-27230
Santa-Cruz
35 kD
Residual GAPDH after stripping at 37 kD

Precision Plus Protein
All Blue Protein
Standard
Bio-Rad 1610373

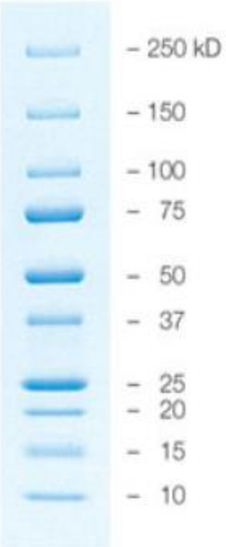

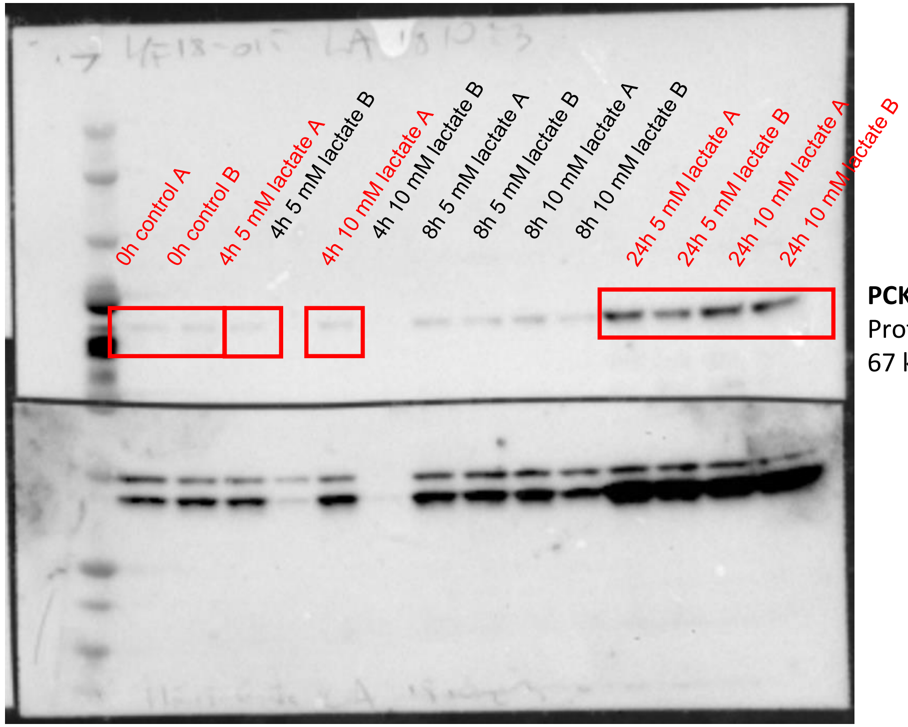

PCK1, 16754-1-AP
Proteintech
67 kD

Raw images of western blot membrane from Figure 3f Part 3

The membrane was cut at 50kD to include more analysed proteins-the antibodies were optimised before the experiments and did not demonstrate unspecific binding

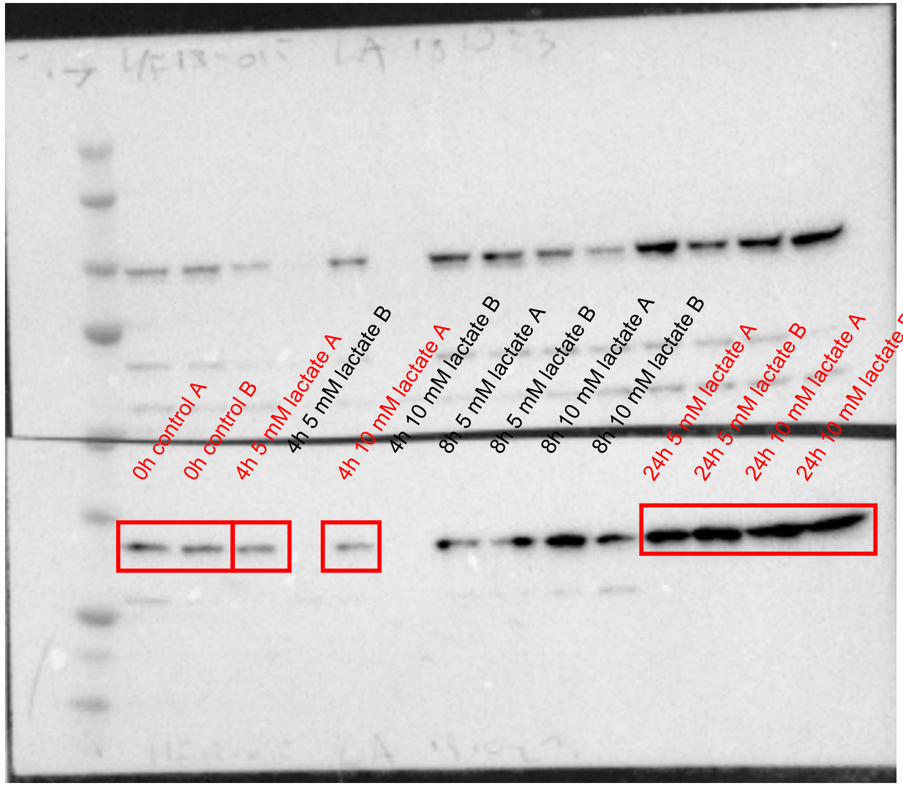

LDHB, ab85319
Abcam
37 kD

Precision Plus Protein
All Blue Protein
Standard
Bio-Rad 1610373

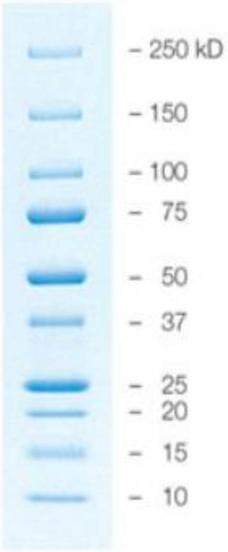

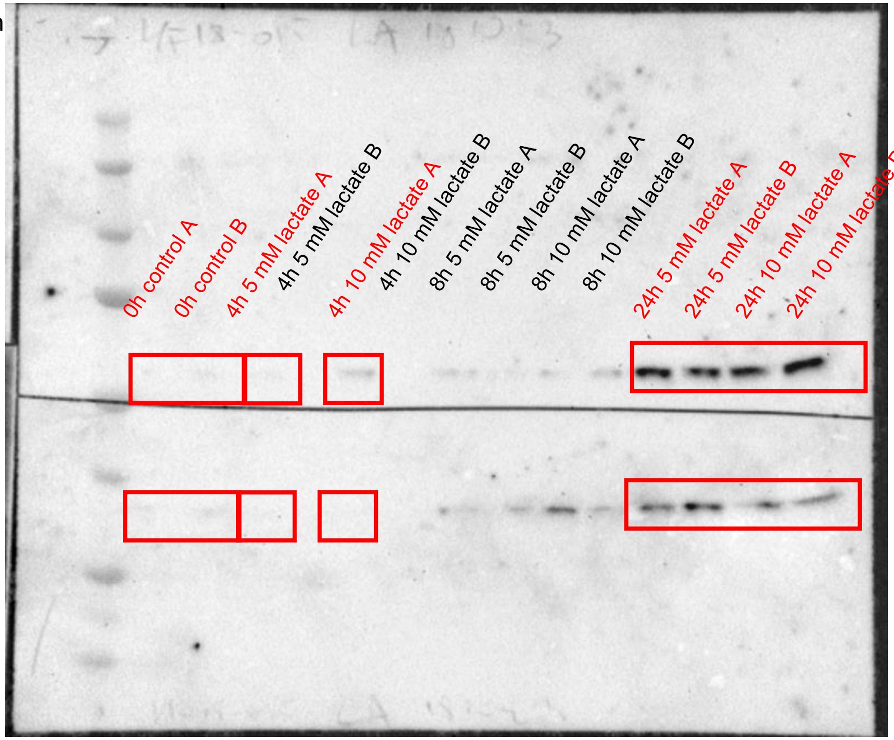

G6PD, sc-373886
Santa Cruz
58 kD

G6Pase, sc-398155
Santa Cruz
36 kD

Raw images of western blot membranes from Figure 4f and Supplementary Figure 3

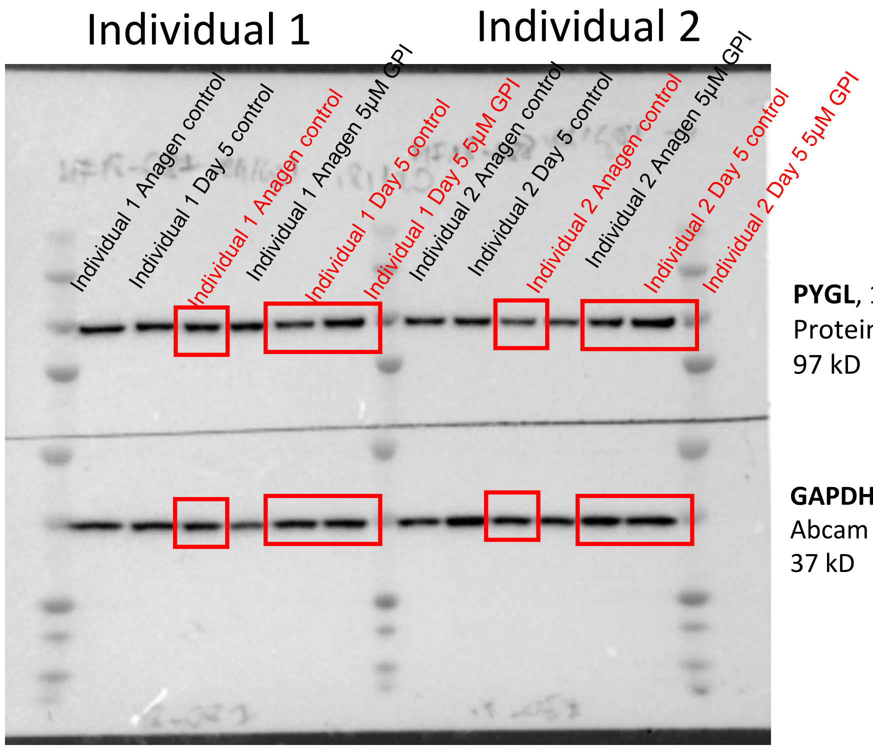

PYGL, 15851-1-
Proteintech
97 kD

GAPDH, ab9485
Abcam
37 kD

Precision Plus Protein All Blue Protein Standard

Bio-Rad 1610373

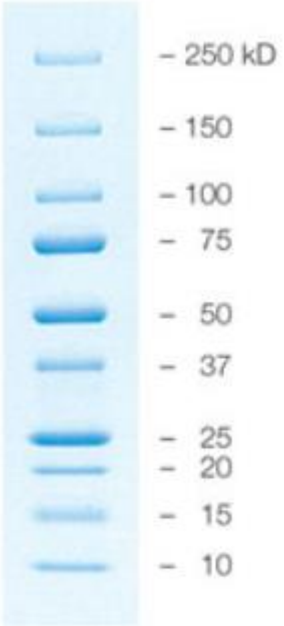

Individual 3

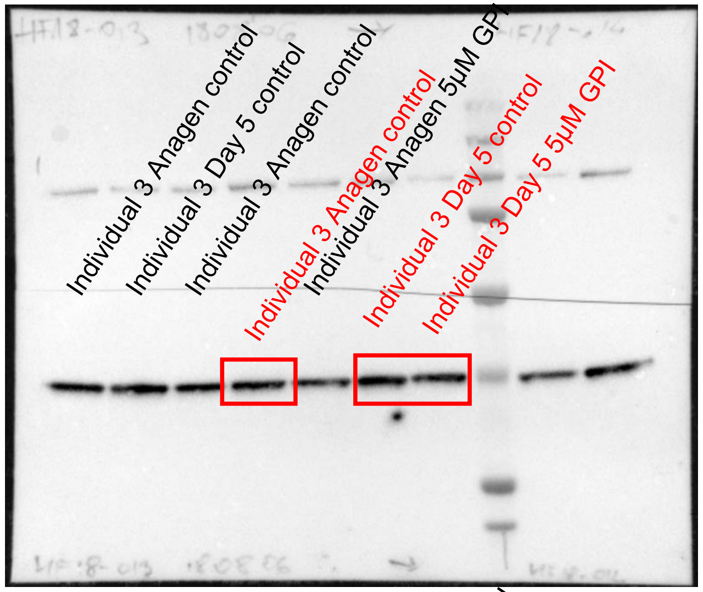

GAPDH, ab9485
Abcam
37 kD

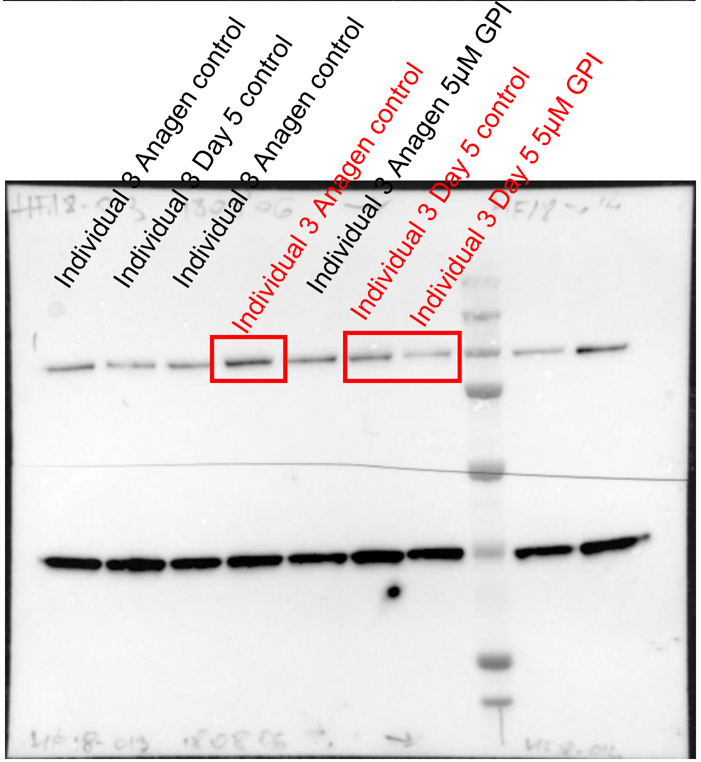

PYGL, 15851-1-AP
Proteintech\
97 kD

The membrane was cut at 50kD to include more analysed proteins-the antibodies were optimised before the experiments and did not demonstrate unspecific binding

Raw images of western blot membranes from Supplementary figure 2-part 1

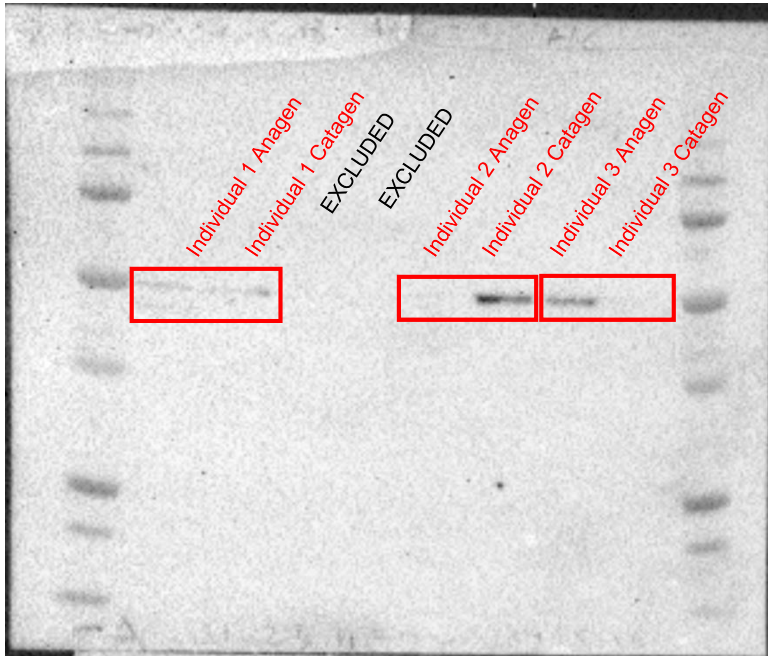

Glut 1, 21829-1-AP
Proteintech
45-55 kD

Precision Plus Protein All Blue Protein Standard

Bio-Rad 1610373

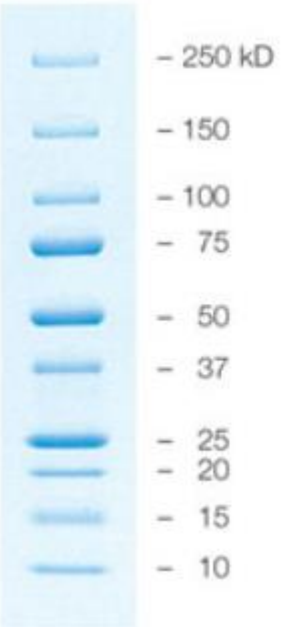

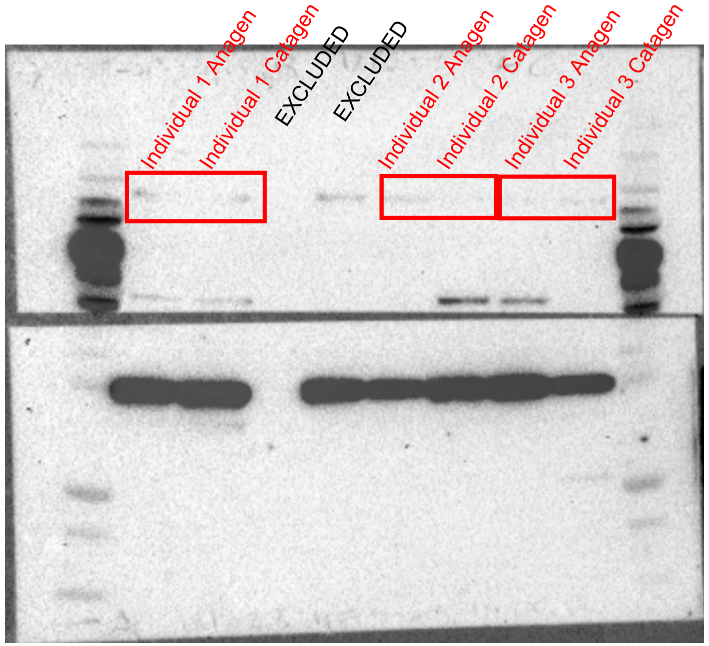

GYS1, 10566-1-AP
Proteintech
84 kD
Residual Glut1 after stripping at 50 kD

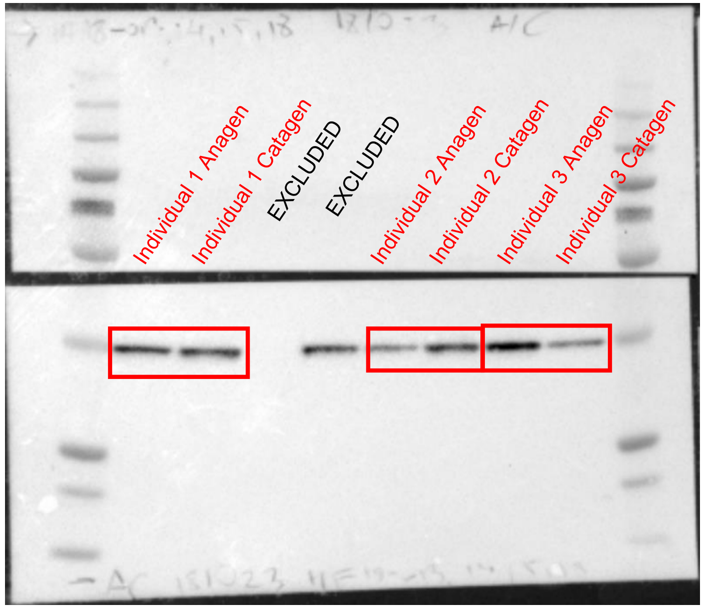

GAPDH, ab9485
Abcam
37 kD

The membrane was cut at 50kD after Glut1 to include more analysed proteins-the antibodies were optimised before the experiments and did not demonstrate unspecific binding

Raw images of western blot membrane from Supplementary figure 2

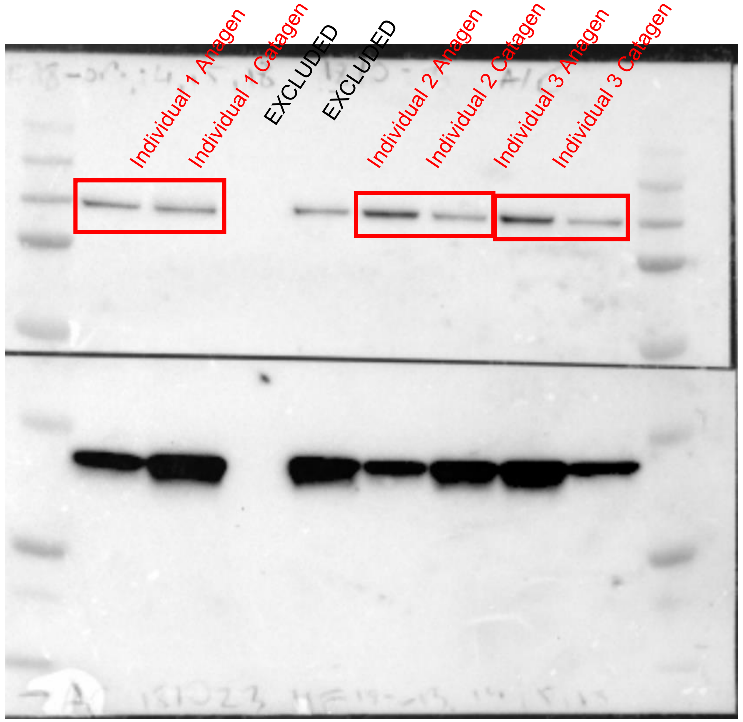

PYGL, 15851-1-
Proteintech
97 kD

Precision Plus Protein All Blue Protein Standard

Bio-Rad 1610373

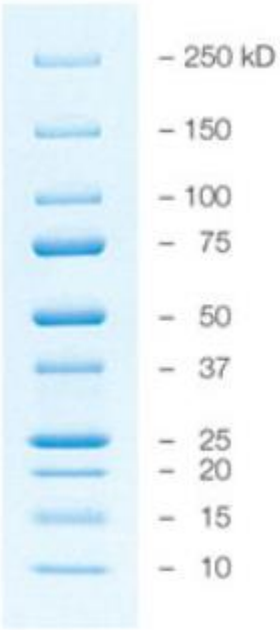

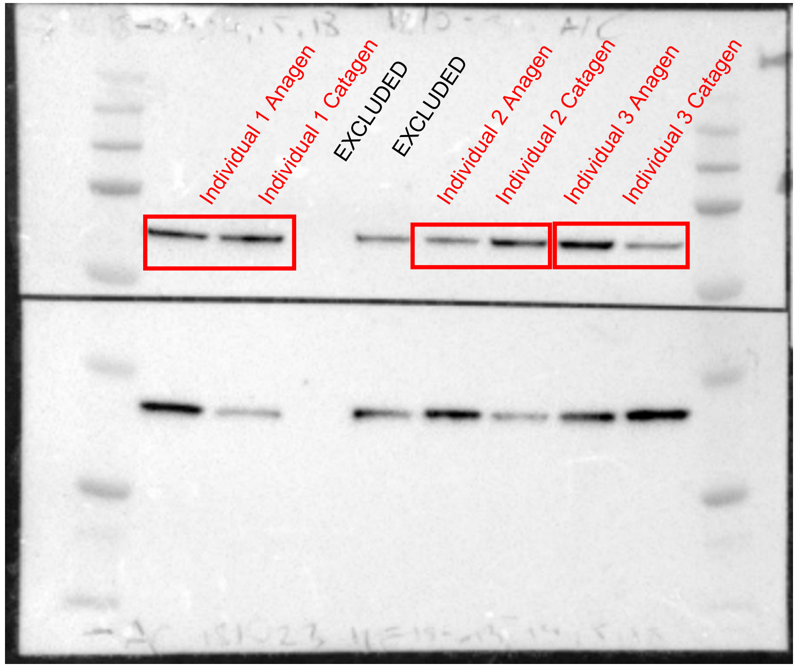

PGM1, ab192876
Abcam
61 kD

The membrane was cut at 50kD after Glut1 to include more analysed proteins-the antibodies were optimised before the experiments and did not demonstrate unspecific binding
