# Supplementary material for: Human hair follicles operate an internal Cori cycle and modulate their growth via glycogen phosphorylase
Source: Sci Rep. 2021 Oct 21;11:20761. doi: 10.1038/s41598-021-99652-8 (PMC8531296; doi:10.1038/s41598-021-99652-8)
Supplement: Supplementary file 2 — Supplementary Information 2. [file 41598_2021_99652_MOESM2_ESM.pdf]

Supplementary figures

**Human hair follicles operate an internal Cori cycle and modulate their growth
via glycogen phosphorylase**

Katarzyna Figlak[1], Greg Williams[2], Marta Bertolini[3], Ralf Paus[3,4,5] and Michael P Philpott[1]*

[1]Centre for Cell Biology and Cutaneous Research, Blizard Institute, Barts & The London School of Medicine and Dentistry, Queen Mary College University of London, UK; [2]Farjo Hair Institute, London, UK; [3]Monasterium Laboratory, Münster, Germany; [4]Dr. Phillip Frost Department of Dermatology and Cutaneous Surgery, University of Miami Miller School of Medicine, Miami, FL, USA; [5]Dermatology Research Centre, University of Manchester, and NIHR Biomedical Research Centre, Manchester, UK

Supplementary Figure 1

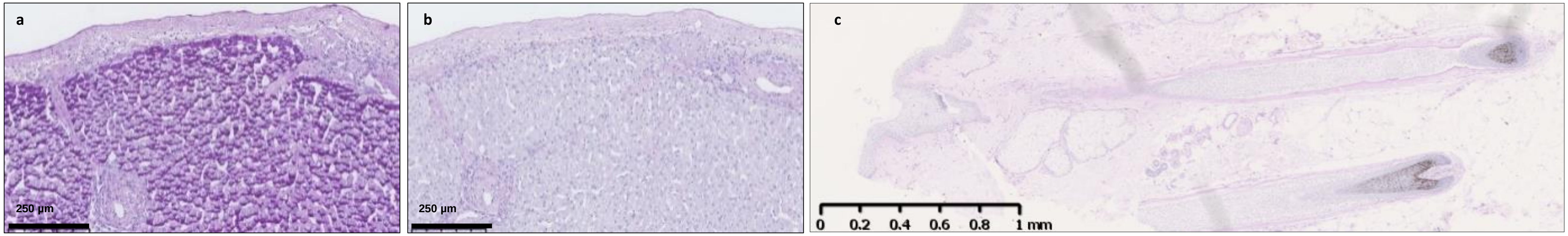

Supplementary Data Figure 1:

Supplement to Figure 1a-h.

Human liver was used as a positive control (a). Specificity of PAS staining for glycogen was shown by digestion of human liver (b) and scalp skin (c) with 0.5% porcine α-amylase (diastase).

Supplementary Figure 2

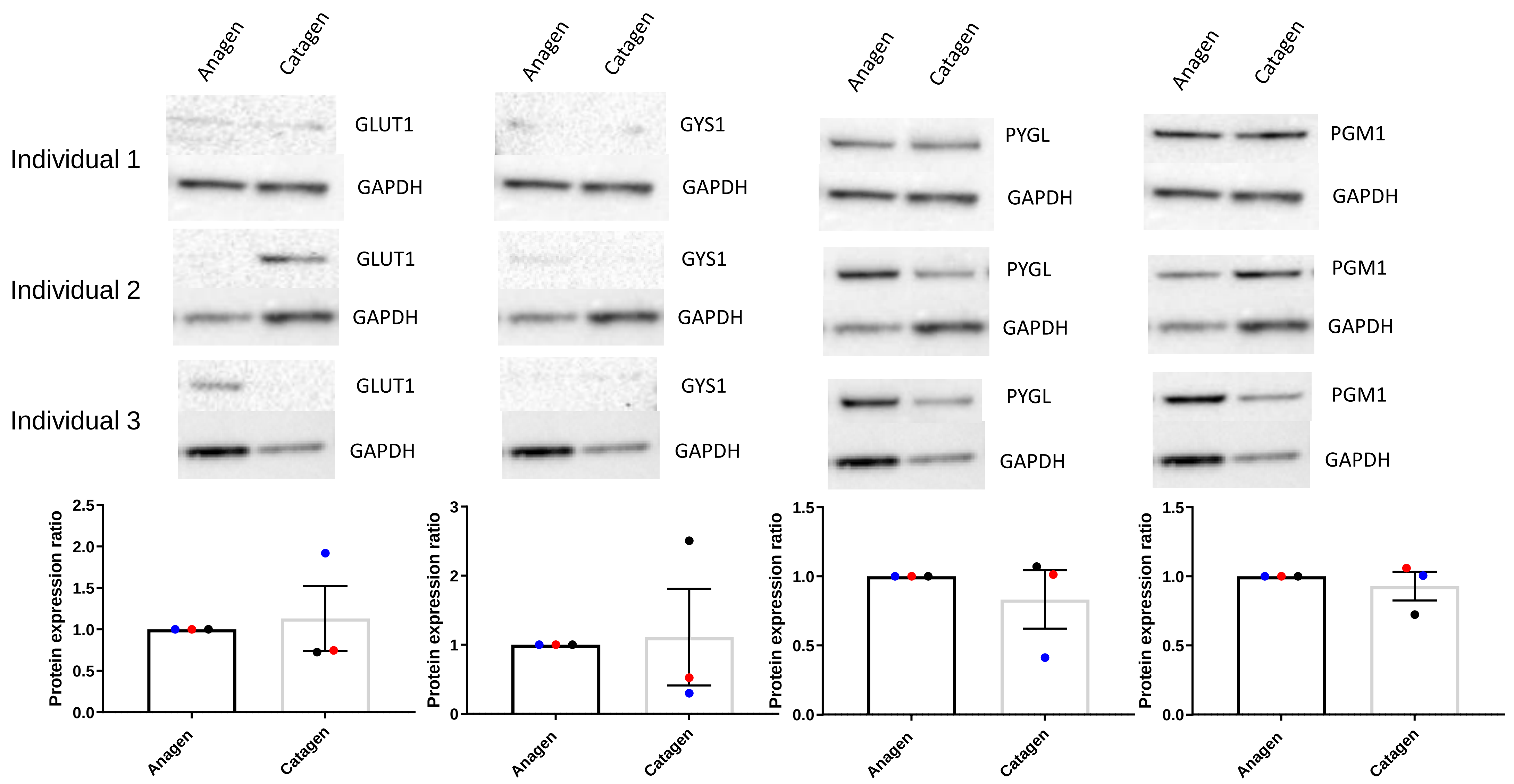

Supplementary Data Figure 2:

Quantified ratios protein expression analysed by western blot and representative images from western blot analysis for GLUT1, GYS1, PYGL and PGM1. GAPDH was used as a loading control. Densitometry measurement carried out in ImageJ software, 3-4 HFs per sample, N=3 (paired two-tailed t-test).

Supplementary Figure 3

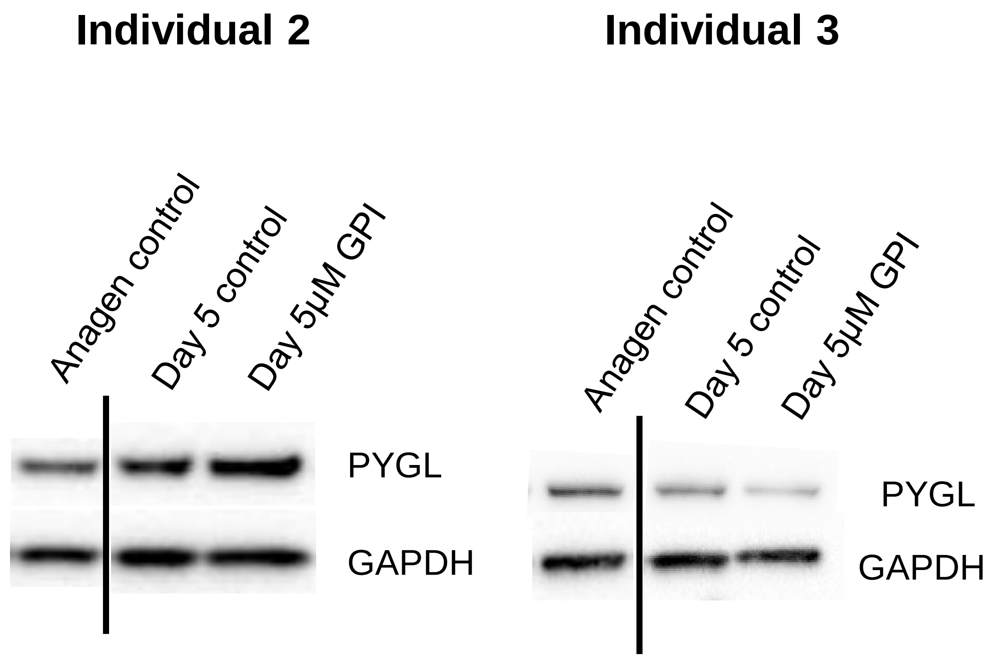

Supplementary Data Figure 3

Supplement to Figure 3f

Western Blot analysis of PYGL expression in HF lysates, 3-4 HFs per sample. GAPDH used as a loading control.
